# A novel c-di-GMP signal system regulates biofilm formation in *Pseudomonas aeruginosa*

**DOI:** 10.15698/mic2020.06.720

**Published:** 2020-04-23

**Authors:** Gukui Chen, Haihua Liang

**Affiliations:** 1 Key Laboratory of Resources Biology and Biotechnology in Western China, Ministry of Education, College of Life Sciences, Northwest University, Xi'an, ShaanXi, 710069, China.

**Keywords:** c-di-GMP, SiaD, Biofilm, Pseudomonas aeruginosa

## Abstract

The bacterial second messenger cyclic-di-GMP (c-di-GMP) controls biofilm formation and other phenotypes relevant to pathogenesis. The human pathogen *Pseudomonas aeruginosa* encodes 17 diguanylate cyclase (DGCs) proteins which are required for c-di-GMP synthesis. Therefore, the c-di-GMP regulatory system in *P. aeruginosa* is highly sophisticated. SiaD, one of the DGC enzymes, is co-transcribed with SiaA/B/C and has been shown to be essential for bacterial aggregate formation in response to environmental stress. However, the detailed function of this operon remains unknown. In our recent paper (Chen *et al.*, doi: 10.15252/embj.2019103412), we have demonstrated that the *siaABCD* operon encodes a signaling network that regulates biofilm and aggregate formation by modulating the enzymatic activity of SiaD. Among this signaling system, SiaC interaction with SiaD promotes the diguanylate cyclase activity of SiaD and subsequently facilities the intracellular c-di-GMP synthesis; SiaB is a unique protein kinase that phosphorylates SiaC, whereas SiaA phosphatase can dephosphorylate SiaC. The phosphorylation state of SiaC is critical for its interaction with SiaD, which will switch on or off the DGC activity of SiaD. This report unveils a novel signaling system that controls biofilm formation, which may provide a potential target for developing antimicrobial drugs.

The intracellular messenger cyclic dimeric (3`-5`) GMP (cyclic di-GMP or c-di-GMP) regulates various aspects of the physiology of diverse environmental and pathogenic bacteria. One of the diguanylate cyclases (DGCs), SiaD, is essential for cell aggregation in response to the toxic detergent sodium dodecylsulfate (SDS) in *P. aeruginosa*. SiaD is co-transcribed with SiaA/B/C, however, the detailed functions of this operon and the relationships among the four genes remain elusive. Additionally, SiaD is required for the macroscopic aggregates formation of *P. aeruginosa* when grown in medium with SDS as the sole carbon source but no aggregate forms when succinate is used as the sole carbon source. Thus, a key question remains to be addressed in this issue: which is the molecular mechanism underlying the activation/modulation of SiaD function? In this scenario, we have recently demonstrated that the direct interactions among SiaC, SiaA, SiaB and SiaD consist of a signaling network that regulates biofilm formation by modulating the intracellular c-di-GMP levels in *P. aeruginosa*.

## THE BINDING PARTNER OF SiaC

Phenotypic assays revealed that SiaC positively regulates biofilm and aggregates formation through SiaD. Bacterial two-hybrid and GST pull down assays showed the direct interaction between SiaC and SiaD. An *in vitro* catalytic assay demonstrated that SiaC promoted the DGC activity of SiaD. Therefore, SiaC acts as a binding partner to modulate the activity of SiaD via direct, physical interaction.

## THE KINASE SiaB

SiaB negatively regulates biofilm formation, which is dependent on SiaC and SiaD. The bacterial two-hybrid and GST pull-down assays showed a direct interaction between SiaC-SiaB but not for SiaB-SiaD, suggesting that SiaB regulates biofilm formation via SiaC. More importantly, our data demonstrate that SiaB encodes an unusual kinase, which specifically phosphorylates Thr-68 of SiaC. Moreover, we solved the crystal structure of the SiaB-SiaC complex and identified residues specifically involved in SiaB catalysis, SiaB-SiaC interaction and SiaB dimer formation, respectively.

## THE FUNCTION OF SiaA

SiaA is predicted to be a membrane protein that contains a PP2C-like phosphatase domain. The function of SiaA and SiaC are interdependent during biofilm formation. *In vitro* radioactive phosphorylation assays revealed that SiaA is able to dephosphorylate SiaC at the Thr-68 residue, confirming the phosphatase activity of SiaA.

## PHOSPHORYLATION OF SiaC AT THR-68

GST pull-down and surface plasmon resonance assays demonstrated that phosphorylation of Thr-68 of SiaC prevents SiaC-SiaD interaction, and thus SiaD activation. Therefore, the antagonistic activity of SiaB and SiaA at Thr-68 of SiaC modulates SiaC-SiaD interaction, and consequently regulates the activation of SiaD.

**Figure 1:**
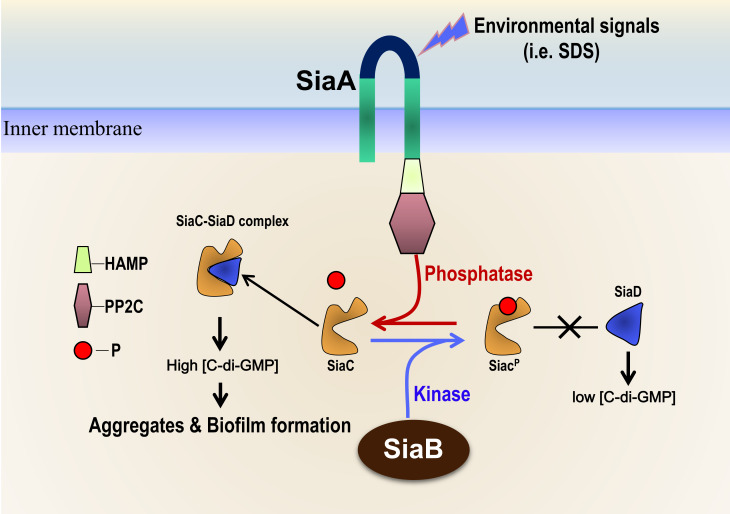
FIGURE 1: Proposed model of the SiaA/B/C/D signaling network involved in regulation of biofilm and aggregate formation. Under normal conditions (such as in rich media), the protein kinase SiaB dominates and maintains most SiaC molecules phosphorylated, which precludes the promotion of the DGC activity of SiaD. Under stress conditions (such as SDS exposure), SiaA phosphatase is activated and most SiaC remains dephosphorylated; in this situation, the dephosphorylated SiaC interacts with SiaD to stimulate the DGC activity of SiaD and subsequently produces sufficient c-di-GMP, which leads to the formation of aggregates and biofilms.

Taken together, we identified a novel signaling network that regulates biofilm formation by modulating the activity of SiaD, wherein SiaC switches the DGC activity of SiaD “on” or “off” after receiving an upstream signal from SiaB or SiaA. *P. aeruginosa* may use this signaling network to rapidly respond and adapt to environmental changes (**Figure 1**). **U**nder normal conditions (such as in rich media), the kinase SiaB maintains SiaC phosphorylation, preventing SiaC-SiaD interaction and the DGC activity of SiaD. Under stress conditions (such as exposure to SDS), SiaA phosphatase is activated by an unknown signal and most SiaC molecules remain dephosphorylated. The dephosphorylated SiaC interacts with SiaD, which stimulates the activity of SiaD and subsequently c-di-GMP production, thus promoting biofilm and aggregates formation.

## PERSPECTIVE

We demonstrated that the binding partner SiaC activates the DGC activity of SiaD via direct interaction and it seems unlikely that direct interaction with SiaC stabilizes SiaD or represses the allosteric inhibition by c-di-GMP. Therefore, the underlying mechanism by which SiaC-SiaD interaction promotes the DGC activity remains elusive. Modulation of activity has been reported for several DGCs, including WspR and PleD. Oligomerization is involved in the modulation of WspR activity through the switch from an active to a product-inhibited dimer via tetrameric assembly. Additionally, modulation of the active aspartate residue with the phosphoryl analog BeF_3_^-^ leads to stabilization of the PleD dimer, which is the catalytically active form. Therefore, we hypothesize that the SiaC-SiaD interaction may alter the conformation of SiaD to promote its binding to GTP or facilitate its catalysis. However, further studies are needed to determine the potential mechanisms for SiaD activation.

